# Fine-Tuning Stomatal Movement Through Small Signaling Peptides

**DOI:** 10.3389/fpls.2019.00069

**Published:** 2019-01-30

**Authors:** Xinyun Qu, Bing Cao, Jingke Kang, Xuening Wang, Xiangyu Han, Wenqian Jiang, Xiong Shi, Luosha Zhang, Langjun Cui, Zhubing Hu, Yonghong Zhang, Guodong Wang

**Affiliations:** ^1^National Engineering Laboratory for Resource Development of Endangered Crude Drugs in Northwest China, Key Laboratory of Medicinal Resources and Natural Pharmaceutical Chemistry, Ministry of Education, College of Life Sciences, Shaanxi Normal University, Xi’an, China; ^2^State Key Laboratory of Cotton Biology, Department of Biology, Institute of Plant Stress Biology, Henan University, Kaifeng, China; ^3^Laboratory of Medicinal Plant, School of Basic Medicine, Hubei University of Medicine, Shiyan, China

**Keywords:** abscisic acid, receptor-like kinase, small signaling peptide, stomatal movement, stress

## Abstract

As sessile organisms, plants are continuously exposed to a wide range of environmental stress. In addition to their crucial roles in plant growth and development, small signaling peptides are also implicated in sensing environmental stimuli. Notably, recent studies in plants have revealed that small signaling peptides are actively involved in controlling stomatal aperture to defend against biotic and abiotic stress. This review illustrates our growing knowledge of small signaling peptides in the modulation of stomatal aperture and highlights future challenges to decipher peptide signaling pathways in guard cells.

## Introduction

Plants are confronted with various biotic and abiotic stress conditions during their life cycle. Plant stomatal pores, consisting of a pair of guard cells, are dynamic structures that open and close to modulate gas exchange for photosynthesis and transpirational water loss, thus allowing plants to respond appropriately to diverse environmental stimuli (Blatt et al., 2017). In addition, stomata are also the major sites for bacterial entry. Therefore, guard cells-mediated stomatal opening and closure serves as an useful strategy to defend against pathogen attack (Kim et al., 2010; Blatt et al., 2017). Stomatal opening involves the activation of H^+^-ATPases in the plasma membrane of guard cells. The activation of H^+^-ATPases results in membrane hyperpolarization that induces K^+^ uptake via activation of inward K^+^ rectifying channels, thereby generating an increased turgor in guard cells to induce stomata opening. Stomatal closure requires the inhibition of H^+^-ATPase and the activation of anion channels, which synergistically cause membrane depolarization and K^+^ efflux through activation of K^+^ outwardly rectifying channels (Kim et al., 2010; Daszkowska-Golec and Szarejko, 2013). Additionally, the elevation of cytosolic Ca^2+^ accompanies stomatal closure (Kim et al., 2010). The molecular mechanisms underlying stomatal opening and closure have been extensively studied (Kim et al., 2010; Daszkowska-Golec and Szarejko, 2013; Murata et al., 2015). Notably, conventional phytohormones, including abscisic acid (ABA), brassinosteroids, striglactones, salicylic acid (SA), and jasmonic acid (JA), have been found to play pivotal roles through their coordination with key transcription factors to control stomatal movement in response to fluctuating environmental conditions (Daszkowska-Golec and Szarejko, 2013; Murata et al., 2015; Lv et al., 2018; Zhang et al., 2018). In this regard, ABA serves as a central hub that integrates various signaling pathways to cause stomatal closure by activating S-type anion channels, SLOW ANION CHANNEL-ASSOCIATED 1 (SLAC1) and its homolog SLAC1 HOMOLOG3 (SLAH3) (Kim et al., 2010; Roelfsema et al., 2012). Additionally, OPEN STOMATA1 (*OST1*), an essential gene for ABA-induced stomatal closure, has been shown to phosphorylate and activate SLAC1 in guard cells (Geiger et al., 2009; Lee et al., 2009; Vahisalu et al., 2010).

**FIGURE 1:**
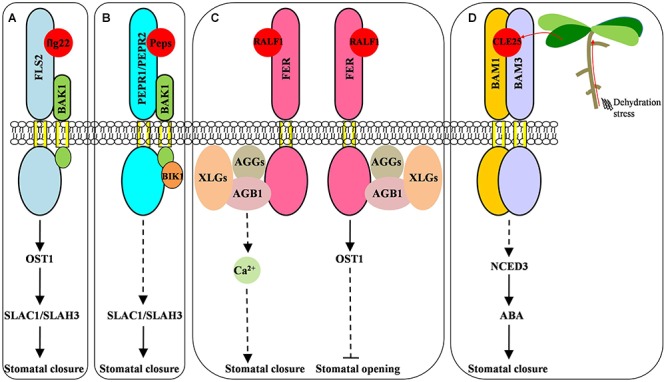
Small signaling peptides-mediated stomatal movement. **(A)** Pathogen-derived flg22 induced stomatal closure and stimulated the anion channels in an OST1-dependent manner. Upon flg22 perception, the FLS2 receptor interacts with the coreeptor BAK1 to activate OST1 which could phosphorylate the anion channel SLAC1 in guard cells. Additionally, the flg22-induced cytosolic Ca^2+^ elevation could activate SLAC1 and SLAH3. The activation of anion channels SLAC1 and SLAH3 release anions into the guard cell wall, thereby depolarizing the plasma membrane to induce stomatal closure. **(B)** The AtPeps-PEPR signaling pathway close stomata by activating guard cell anion channels SLAC1/SLAH3 in an OST1-independent manner. **(C)** The RALF1-FER signaling module mediated stomatal opening and closure through distinct pathways. All of the G protein subunits, except GAP1, are involved in the induction of stomatal closure and the inhibition of stomatal opening triggered by RALF1. RALF1-FER signaling may trigger cytosolic Ca^2+^ elevation, which induces stomatal closure. OST1 is only involved in the RALF1 inhibition of stomatal opening, but not in the RALF1 promotion of stomatal closure. **(D)** The CLE25 peptide-mediated root-to-shoot signaling stimulated the stomatal closure under dehydration stress. Dehydration stress induces *CLE25* expression in roots. The resultant synthesized peptide is transported to the leaves, where it binds BAM1/BAM3 and subsequently stimulates ABA accumulation through activating *NCED3* expression; this in turn induces stomatal closure. Dashed lines represent missing step(s)/component(s) that are yet-undetermined in the signaling pathway.

Numerous small signaling peptides have been found in many plant species, although most of them are yet to be functionally characterized (Czyzewicz et al., 2013). Small signaling peptides contribute substantially to plant growth and development (Wang and Fiers, 2010; Murphy et al., 2012). In addition, considerable advances have been also achieved towards our understanding of interactions between small signaling peptides and environmental stimuli (Wang et al., 2016). Particularly, it was recently found that small signaling peptides are actively implicated in controlling stomatal aperture to defend against biotic and abiotic stress (Li et al., 2014; Takahashi et al., 2018; Yu et al., 2018; Zheng et al., 2018). The Arabidopsis receptor-like kinase FLAGELLIN-SENSITIVE2 (FLS2) recognizes flg22, a 22-amino acid peptide derived from the bacterial flagellin protein (Monaghan and Zipfel, 2012). Upon flg22 perception by FLS2, which interacts with the coreceptor BRI1-ASSOCIATED KINASE 1 (BAK1), a series of physiological events occurs sequentially, including a transient elevation of cytosolic calcium and the production of reactive oxygen species (ROS) which could ultimately induce stomatal closure as a defense strategy to prevent bacterial invasion (Figure 1A; Melotto et al., 2006; Li et al., 2014; Deger et al., 2015). Mechanistically, the flg22-stimulated receptor complexes activate OST1, which phosphorylates the anion channel SLAC1 in guard cells. In addition, flg22-induced cytosolic Ca^2+^ elevation could activate SLAC1 and SLAH3. The activation of anion channels SLAC1 and SLAH3 releases anions into the guard cell wall, thereby depolarizing the plasma membrane to induce stomatal closure (Figure 1; Deger et al., 2015). Recently, more studies have provided novel mechanistic insights into small peptide-mediated signaling and its modes of action in stomatal movement (Takahashi et al., 2018; Yu et al., 2018; Zheng et al., 2018). Here, we review recent advances in cell-cell communication through small signaling peptides and their cognate receptors, with a focus on stomatal movement.

## Plant Elicitor Peptides (Peps) Close Stomata by Activating Guard-Cell Anion Channels in an Ost1-Independent Manner in Arabidopsis

It has been known that Peps, a family of damage/danger-associated molecular patterns (DAMPs), contribute to plant defense against pathogen attack and abiotic stress through perceiving by two closely-related receptor-like kinases, PEPR1 and PEPR2 (Yamaguchi et al., 2006, 2010). Enhanced bacterial growth was observed in *pepr1 pepr2* double mutant plants sprayed with *Pseudomonas syringae* pv *tomato* (*Pst*) DC3000, but not in plants infiltrated with bacteria, indicating that PEPR-mediated stomatal defense may be achieved by inducing stomatal closure as a mechanism to restrict bacterial entry. Indeed, *in vitro* application of AtPeps significantly induced stomatal closure in wild-type plants, but not in *pepr1 pepr2* mutants, underscoring the importance of the AtPeps-PEPR signaling module in the induction of stomatal closure, and indicating that AtPeps-induced stomatal closure is PEPR-dependent (Figure 1B; Zheng et al., 2018). Further investigation revealed that SLAC1 and SLAH3 are required for the AtPep1-induced stomatal closure based on the result that *slac1 slah3* double mutants, but not *slac1* or *slah3* single mutants, are impaired in the AtPep1-induced stomatal closure (Zheng et al., 2018). Consistently, guard cells of *slac1 slah3* double mutants had much smaller S-type anion currents compared with the wild-type or their single mutant plants. However, disruption of OST1, a central regulator in stomatal closure, did not impair the anion channel activity or AtPep1-induced stomatal closure (Zheng et al., 2018), suggesting that, unlike the flg22-FLS2 pathway, OST1 is dispensable for AtPep1-induced stomatal closure (Melotto et al., 2006; Zheng et al., 2018). BOTRYTIS-INDUCED KINASE1 (BIK1), a coreceptor of PEPR, was also implicated in AtPep1-induced stomatal response as *bik1* mutants failed to respond to AtPep1 in stomatal closure assays (Zheng et al., 2018). Together, these results indicate that the AtPeps-PEPR signaling module exploits a unique mechanism for stomatal closure promotion through SLAC1 and SLAH3 activation in an OST1-independent manner. Nevertheless, it remains largely unknown that how the AtPeps-PEPR signaling pathway stimulates stomatal closure. Thus, further experiments, such as the identification of critical intermediate component(s), detection of physical interaction(s) among different components, and determination of the order of events, will facilitate the elucidation of the mechanism of AtPeps-PEPR-induced stomatal closure.

## Rapid Alkalinization Factor 1 (RALF1)-Mediated Stomatal Opening and Closure Through Distinct Pathways

The RALF family, comprising 35 members, is a group of cysteine-rich peptides that control alkalization and cell expansion (Haruta et al., 2014; Murphy and De Smet, 2014; Stegmann et al., 2017). RALF1 is the most well-studied member of the RALF family. RALF1 has been reported to directly bind the receptor-like kinase FERONIA (FER) and stimulate phosphorylation of FER and other proteins (Haruta et al., 2014). Heterotrimeric guanine nucleotide-binding (G) proteins are composed of Gα, Gβ, and Gγ subunits, and function in various biological processes including growth and development as well as stress resilience in Arabidopsis (Trusov and Botella, 2016). Recently, FER was identified as one of the Gβ subunit (AGB1)-associated proteins through co-immunoprecipitation and mass spectrometry (Yu et al., 2018). In combination with the fact that FER and G proteins are involved in the modulation of the guard-cell ABA response, it thus raises the hypothesis that RALF1 is also involved in the stomatal response. Indeed, it was found that RALF1 inhibited stomatal opening and promoted stomatal closure, while both RALF1 effects were completely abolished in *fer* mutants, indicating that RALF1-regulated stomatal aperture is dependent on the FER receptor (Figure 1B; Yu et al., 2018). In addition, two independent *agb1* mutants failed to respond to RALF1 peptides in stomatal opening and closure assays, indicating that AGB1 is necessary to transduce the RALF1 signal in stomatal movement. Likewise, disruption of three Gγ subunits (AGG1, AGG2, and AGG3) rendered the *agg* triple mutant insensitive to RALF1 in both stomatal opening and closure (Yu et al., 2018). However, the loss-of-function mutant of the canonical Gα protein GPA1 responded normally to RALF1 application, in a manner similar to wild-type plants with respect to the induction of stomatal closure and the inhibition of stomatal opening. Conversely, disruption of three extra-large Gα subunits (XLG1, XLG2, and XLG3) impaired RALF1-mediated stomatal movement (Yu et al., 2018). Interestingly, OST1 is only involved in RALF1 inhibition of stomatal opening, but not in RALF1 promotion of stomatal closure (Yu et al., 2018). Overall, these results identified a G protein-dependent function for the RALF1-FER signaling module in the modulation of stomatal movement, in which several guard-cell ABA signaling components are also required. Combined with previous studies, it is likely that RALF1 promotes stomatal closure through activation of cytosolic Ca^2+^ signaling in an OST1-independent manner but inhibits stomatal opening in an OST1-dependent manner, suggesting that RALF1 differentially regulates stomatal opening and closure through distinct signaling pathways (Allen et al., 2001; Haruta et al., 2008; Yu et al., 2018). However, the biological relevance of RALF1-mediated stomatal movement remains elusive. FER has been shown to play roles in multiple processes including facilitation of pathogen invasion (Kessler et al., 2010; Stegmann et al., 2017); thus, it is speculated that RALF1-FER signaling may be involved in stomatal immunity. Alternatively, given the fact that FER influenced the modulation of stomatal aperture by ABA, it is possible that RALF1 may crosstalk with ABA to coordinate the stomatal movement (Fan et al., 2008; Yu et al., 2012; Chen et al., 2016).

## The Clavata3/Endosperm Surrounding Region-Related 25 (CLE25) Peptide-Mediated Root-To-Shoot Signaling Modulates Stomatal Closure

In *Arabidopsis thaliana*, the CLE peptide family has been extensively studied, mainly in the context of stem cell homoeostasis (Betsuyaku et al., 2011; Murphy et al., 2012; Czyzewicz et al., 2013). Other than their notable roles in stem cell fate, *CLE* genes have been found to be implicated in a wide-range of biological processes and to mediate plant responses to environmental stimuli (Wang et al., 2016). Intriguingly, it was recently found that the CLE25 peptide serves as a root-to-shoot long-distance signal that results in remote control of the stomatal closure mediated by ABA during dehydration stress (Figure 1D) (Takahashi et al., 2018). Specifically, *CLE25* is expressed in the vascular tissues of roots and leaves, and its expression was rapidly elevated only in roots following dehydration (Takahashi et al., 2018). Most importantly, among 27 chemically synthesized CLE peptides, only CLE25 application to roots could stimulate the foliar expression of a key ABA biosynthetic enzyme gene, *NINE*-*CIS*-*EPOXYCAROTENOID* *DIOXYGENASE* *3* (*NCED3*), and thus enhance ABA accumulation in leaves, whereas *NCED3* expression is heavily suppressed in CLE25 knockout mutants under dehydration stress conditions (Takahashi et al., 2018). CLE25 application to both roots and leaves effectively induced stomatal closure. Examination of the CLAVATA1/BARELY ANY MERISTEM (CLV1/BAM) receptor-like kinase family identified that BAM1 and BAM3 are required for CLE25-mediated responses, based on the result that enhanced *NCED3* expression and ABA accumulation were abolished in the *bam1 bam3* double mutant following dehydration (Takahashi et al., 2018). Additionally, CLE25 application did not induce *NCED3* expression in leaves of grafted plants in which the *bam1 bam3* shoot was grafted to the rootstock of either the wild-type plant or the double mutant itself. In contrast, CLE25 application enhanced leaf *NCED3* expression in grafted plants in which the WT shoot was grafted to either the wild-type rootstock or the *bam1 bam3* rootstock (Takahashi et al., 2018). The mobility of CLE25 was further confirmed by using a mass-spectrometry technique to identify CLE25 peptides that moved from roots to leaves. Taken together, these results indicated that the root-derived CLE25 peptide, functioning as a long-distance mobile signal, could move to the leaves and bind BAM1/BAM3.

Collectively, it was found that dehydration induces *CLE25* expression in roots, and that the resultant synthesized peptide is transported to the leaves, where it binds BAM1/BAM3 and subsequently stimulates ABA accumulation via activating *NCED3* expression; this in turn induces stomatal closure. However, many questions remain to be addressed concerning steps from dehydration-induced *CLE25* expression to elevated ABA-induced stomatal closure. For instance, how is the CLE25 peptide transported? Given its molecular features, it is likely that transportation of the CLE25 peptide is considerably slower relative to that of conventional phytohormones and hydraulic signals. It thus raises the question how the rapid stomatal closure is achieved with multiple steps through long-distance CLE25 signaling by affecting ABA biosynthesis. In addition, it would be of interest to establish the regulatory network of hydraulic signals, ABA, and CLE25 peptides in the orchestration of stomatal closure. It was also reported that water shortage could result in increased xylem tension, which can act as a rapid signal to induce foliar ABA synthesis (Christmann et al., 2013). It is likely that CLE25 acts together with these signals to regulate stomatal aperture, as it was observed that *cle25* knockout mutants exhibited increased water loss following a 10-min dehydration stress (Takahashi et al., 2018). Moreover, how the recognition of the CLE25 signal by BAM/BAM3 could induce *NCED3* expression remains unknown. ABA acts as a mediator in the CLE25-mediated remote fine-tune stomatal closure under water-deficit conditions in plants. Therefore, future studies will also be necessary to elucidate whether any guard cell ABA-signaling components, such as OST1, ROS, and/or SLAC1/SLAH3, are required for the remote CLE25-mediated stomatal response.

## Concluding Remarks

Accumulating data reveals that small signaling peptides are implicated in the modulation of stomatal aperture (Figure 1). The studies summarized here represent a significant step towards understanding small signaling peptide-mediated stomatal responses and stress acclimation. As discussed, respectively, questions persist regarding the stomatal response stimulated by each aforementioned small signaling peptide. In a broader context, the identification of critical intermediate component(s), examination of physical interaction(s), and phosphorylation regulation of different components, and determination of the sequence of events, are all undoubtedly essential to further elucidate the underlying mechanisms of the stomatal movement mediated by these small signaling peptides. The regulatory network comprising various signaling molecules that coordinate the stomatal response remains unknown. Additionally, a number of studies have suggested the existence of crosstalks of signaling peptides with phytohormones and external stimuli (Wang et al., 2016). In this regard, the aforementioned small signaling peptides are presumably implicated in responding to environmental stimuli and are integrated with phytohormones (e.g., ABA) to modulate stomatal aperture. Nevertheless, the interconnection of these signaling peptides in mediating stomatal movement remains elusive. To date, very few peptide-receptor pairs in the fine-tuning of stomatal movement have been characterized. However, given the complexity of the signaling and the number of predicted peptides, it is expected that more pairs will be identified in the future. Indeed, many small signaling peptide-encoding genes have been found to be highly/specifically expressed in guard cells (Adrian et al., 2015). The function of these small signaling peptides in the fine-tuning of the stomatal response needs to be addressed in the future.

## Author Contributions

All authors contributed to the interpretation, discussion of the literatures, and reviewed and helped to polish the final manuscript. XQ, BC, and GW conceived of the design and structure of the article and the figure. GW wrote the paper with inputs from all authors.

## Conflict of Interest Statement

The authors declare that the research was conducted in the absence of any commercial or financial relationships that could be construed as a potential conflict of interest.
